# Genetic polymorphisms associated with nonalcoholic fatty liver disease in Uyghur population: a case-control study and meta-analysis

**DOI:** 10.1186/s12944-018-0877-3

**Published:** 2019-01-15

**Authors:** Wen Cai, Di-hua Weng, Ping Yan, Yu-ting Lin, Zheng-hui Dong, Mailamuguli, Hua Yao

**Affiliations:** 1 School of Nursing, Xinjiang Medical University, Urumqi, Xinjiang, 830054 People’s Republic of China; 2 The Fourth Affiliated Hospital of Xinjiang Medical University, Urumqi, Xinjiang 830054 People’s Republic of China; 3 Department of Clinical Laboratory, maternal and child health hospital of the Xinjiang Uygur Autonomous Region, Urumqi, Xinjiang 830054 People’s Republic of China; 4 The First Affiliated Hospital of Xinjiang Medical University, Urumqi, Xinjiang 830054 People’s Republic of China; 5 Department of Public Health, Xinjiang Medical University, Urumqi, Xinjiang 830054 People’s Republic of China

**Keywords:** Genetic, Case-control, Polymorphisms, NAFLD, Meta-analysis

## Abstract

**Background:**

Polymorphisms have been identified to predispose to NAFLD. Here, we accessed the seven polymorphisms of rs1260326, rs780094 in GCKR, rs2954021 near TRIB1, rs2228603 in NCAN, rs58542926 in TM6SF2, rs12137855 near LYPLAL1, and rs10883437 near CPN1 on NAFLD susceptibility in the Uygur population.

**Material and methods:**

We collected 620 samples (317 NAFLD and 303 controls) for this case-control study. Meta-analysis was performed using Stata Software.

**Results:**

Our data detected that the rs1260326 (T vs. C: OR = 1.27, 95% CI = 1.01–1.59) and rs780094 (T vs. C: OR = 1.30, 95% CI = 1.04–1.63) were significantly associated with the susceptibility to NAFLD in Uygur population. The rs1260326 and rs780094 T/T genotype are significantly associated with soda, egg, and soybean intakes in the consumption group with twice or more in a week. Furthermore, a significant haplotype effect of rs1260326/T- rs780094/T was found (OR = 1.29, 95% CI: 1.03–1.62) compared with CC haplotype. An additional meta-analysis using 4352 cases and 10,168 controls established that rs780094 (OR = 1.21, 95%CI: 1.14–1.28) is significantly associated with NAFLD. Finally, among the 4 case-control studies on rs1260326, including 712 NAFLD and 658 controls, significant associations were found in Asian, liver biopsy, adult and pediatric groups.

**Conclusion:**

Collectively, both our case-control study and meta-analysis confirm a significant association between rs780094 and NAFLD. Additionally, our results suggest Asian-specific, liver biopsy-specific, adult-specific and pediatric-specific associations between the rs1260326 and NAFLD. Moreover, the rs1260326 and rs780094 T/T genotype are significantly associated with food habits, such as soda, egg, and soybean.

**Electronic supplementary material:**

The online version of this article (10.1186/s12944-018-0877-3) contains supplementary material, which is available to authorized users.

## Introduction

Non-alcoholic fatty liver disease (NAFLD), one of the most common forms of chronic liver diseases, is the epidemic hepatic manifestation of the metabolic syndrome with hyperglycemia, dyslipidemia, and subclinical inflammation [1, 2]. It mainly constitutes a risk factor for progression to fatty liver, non-alcoholic steatohepatitis (NASH), fibrosis, cirrhosis and hepatocellular carcinoma [3, 4]. In China, NAFLD is observed in 15–20% of the population in affluent regions of China with the steadily increasing pandemic of obesity and diabetes [5].

In addition to environmental factors, recent studies suggest that genetic factors are involved in the development and progression of NAFLD [6, 7]. The polymorphisms of several genes, such as glucokinase regulatory protein (GCKR), tribbles homolog 1 (TRIB1), neurocan(NCAN), transmembrane 6 superfamily member 2 (TM6SF2), and Lysophospholipase-like 1(LYPLAL1), were reported to be involved in the genetic susceptibility to NAFLD [8–11]. Additionally, a GWAS study has identified that rs10883437 was associated with elevations in alanine transaminase (ALT) [12]. Genetic loci associated with concentrations of liver enzymes in plasma. However, conflicting results regarding its potential correlation with NAFLD were reported [13–16]. And genetic risk factors for NAFLD may differ between different populations. Accordingly, replicating formerly published genetic associations in different populations are necessary to specify the associations of the genetic risk in each population.

Therefore, in this study, we focus on the seven polymorphisms of rs1260326 (p.Leu446Pro), rs780094 in GCKR, rs2954021 near TRIB1, rs2228603 (p.Pro92Ser) in NCAN, rs58542926 (p.Glu167Lys) in TM6SF2, rs12137855 near LYPLAL1, and rs10883437 near CPN1, and assess the associations between these gene polymorphisms and NAFLD risks in the Chinese Uygur population.

## MATERIAS and methods

### Ethics approval of the study protocol

Written informed consent was obtained from all participants. All participants explicitly provided permission for DNA analyses as well as collection of relevant clinical data. This study was approved by the Ethics Committee of Xinjiang Medical University, Urumqi, China. It was conducted according to the standards of the Declaration of Helsinki.

### Study population

Subjects were from a Uygur population who lived in the Xinjiang Uygur Autonomous Region of China. We recruited the NAFLD group from Xinjiang Medical University between since January 2015 and January 2016, and the control group came from the same hospital in the same period. In the NAFLD group, there were 317 Uygur patients, mean age (42.92 ± 9.44) years with features of NAFLD and ultrasonographic (US) examinations. Inclusion criteria were: diagnosed in accordance with the standards set described previously [17]. Exclusion criteria were: (1) alcohol consumption greater than 20 g/day for males or 10 g/day for females; (2) a positive test for hepatitis B antigens or hepatitis C antibodies; (3) refused to participate in trials. In the Control group, there were 303 of healthy Uygur controls, mean age (42.44 ± 10.05) years. Inclusion criteria were: frequency-matched to the NAFLD patients according to sex, age, ethnicity, and area of residence. Exclusion criteria: acute or chronic infection, malignant tumor, autoimmune diseases. In the dietary section, we calculated following items: red meat, soda, egg, vegetables, fruits and soybean. And we divided consumption of these items into following groups: (i) Once or less in a week (ii) Twice or more in a week.

### Clinical characteristics of the study participants

All patients completed the standard test registration form, and disclosed the following data: (1) General information: age, sex, race, Body mass index (BMI). (2) Special test: serum triglyceride (TG), total cholesterol (TC), high-density lipoprotein (HDL), low-density lipoprotein (LDL), serum uric acid (SUA), fasting plasma glucose (FPG), aspartate aminotransferase (AST), alanine aminotransferase (ALT), blood urea nitrogen (BUN), serum creatinine (SCr), Adiponectin (ADP), Retinol-binding protein 4 (RBP4), Cytokeratin 18-M30 (CK18-M30), and Cytokeratin 18-M65 (CK18-M65). The ADP, RBP4, CK18-M30 and CK18-M65 concentrations in serum were determined by ELISA Kit (MultiSciencesBiotech Co., Ltd., China) according to the manufacture’s instruction.

### DNA extraction and genotyping

Genomic DNA was extracted from whole-blood samples using standard procedures (Promega). We used polymerase chain reaction (PCR)–ligase detection reaction (LDR) method to genotype the six polymorphisms. PCR-LDR reactions were performed as described by the manufacturer (Applied Biosystems, Warrington, UK) [18, 19], with technical support from the Shanghai Genesky Biotechnology Company. Briefly, 4.0-μl of PCR product was incubated at 37 °C for 60 min with 2-U shrimp alkaline phosphatase (SAP) and 2-U Exonuclease I (ExoI). Following a 15-min incubation to inactivate the enzymes, 1 ul of digested PCR product was mixed with 5 ul of ready reaction premix, 1 ul of 1.0- UM primer, and 3 ul of dH_2_O. This mixture was placed in the thermal cycler and underwent 25 cycles of 96 °C for 10 s, 50 °C for 5 s, and 60 °C for 30 s. When completed, 0.5-U SAP was added and the reaction mixture was incubated for 60 min. Prior to loading onto the PRISM 310, 10 ul of formamide was added to 1 ul of reaction mixture and samples were heated to 95 °C for 5 min. Finally, the primary data was analyzed by GeneMapper 4.0 (Applied Biosystems, Foster City, CA, USA).

### Publication retrieval and data extraction

**Fig. 1:**
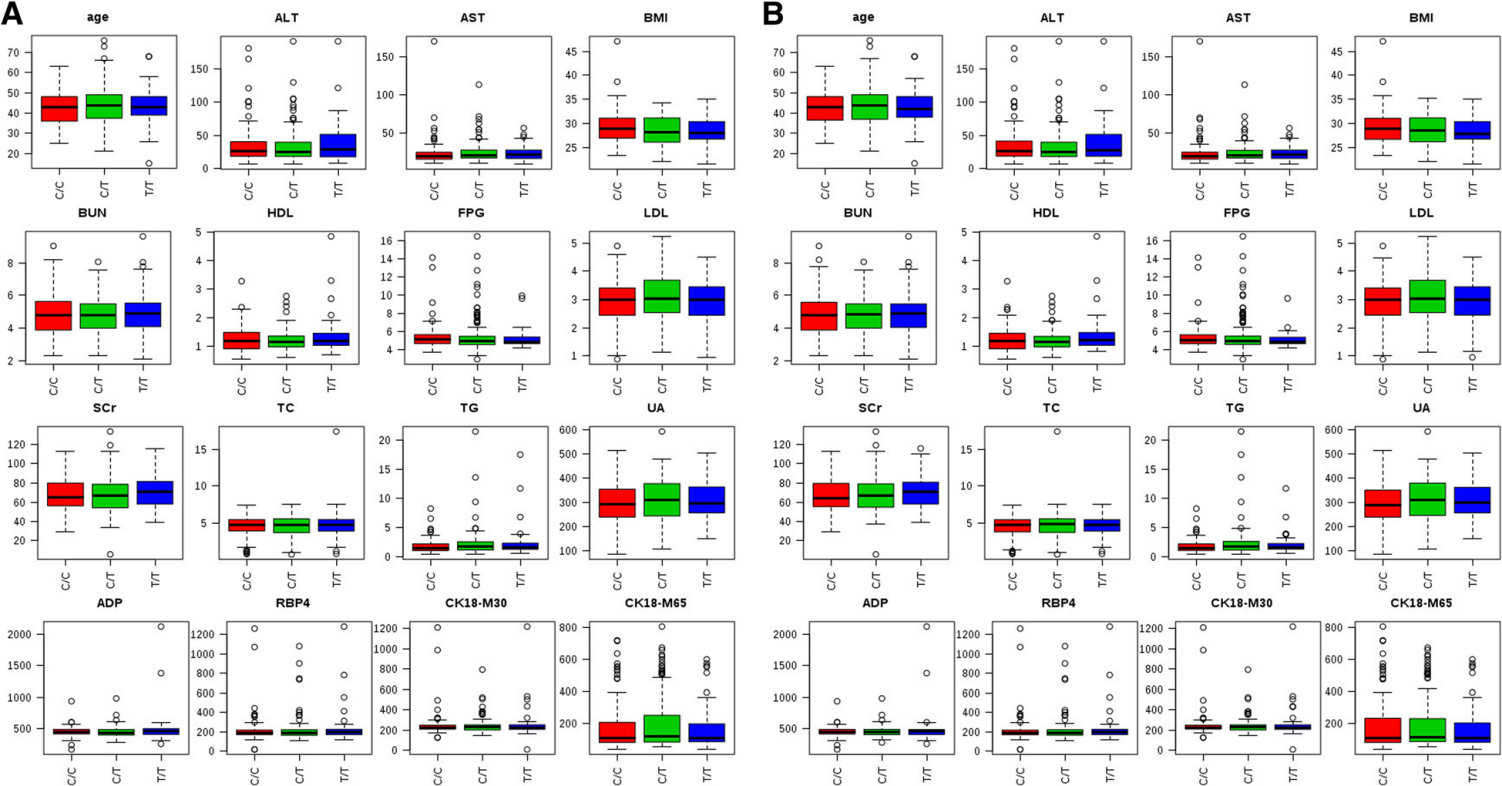
Genotype of rs1260326 (**a**), rs780094 (**b**) and the clinical characteristics of the patients

An independent systematic literature search of studies in all languages to October 2017 across PubMed, EMBASE, Medline, Web of Science, Springer, Cochrane Library, ScienceDirect, and China National Knowledge Infrastructure (CNKI) was conducted. The search terms used were“(GCKR or glucokinase regulatory) and (fatty liver or NAFLD) and (genetic variants or genetic variations or SNPs)”. References from eligible literature were scanned to avoid missing studies. The details on the inclusion criteria included as follow: 1) only the case-control studies on the association between GCKR (rs1260326 and rs780094) and NAFLD were included; 2) the eligible studies must contain enough information for the calculation of odds ratio; 3) the trial should be included the underlying NAFLD as the outcome of study. Two authors (Wen CAI and Ping YAN) independently extracted data included the first author’s name, publication year, country, ethnic group, number of alleles or genotypes and the total number of cases and controls. A total of 44 literatures were identified initially from the search strategy above. After screening titles and abstracts, 34 full texts were then retrieved for details review. At last, the remaining 17 case-control studies were qualified for our meta-analysis (Fig. 1) [8, 13, 14, 20–32].

### Statistical analyses

All continuous variables (e.g., age, BMI, and TG) are presented as means ± standard deviation (S.D.). The difference between the NAFLD and control groups was analyzed using a Student’s t-test, Pearson’s Chi-squared test or the nonparametric Mann–Whitney U tests, as appropriate. The potential relationship of genotypic frequencies of the polymorphisms with the risk of NAFLD was evaluated by the odds ratios (ORs) with their 95% confidence intervals (CIs) from logistic regression models. Meta-analysis was performed using the Stata software (V.11.0; StataCorp, College Station, Texas, USA) set to the fixed-effect or random-effect method according to the heterogeneity. Sensitivity analyses were performed by excluding one study at a time to evaluate the influence of single studies on summary HRs. To evaluate the publication bias, Begg’s funnel plot was assessed [33]. All statistical analyses were analyzed by the Statistical Package for Social Sciences software (SPSS, Windows version, release 22.0; SPSS Inc., Chicago, IL, USA). *P*-values < 0.05 were defined as statistically significant level.

## Results

### Comparison of the clinical data between the patient group and the control group

A total of 620 subjects were enrolled, consisted of 317 NAFLD and 303 healthy controls in this case–control study. Table 1 showed the clinical characteristics of the NAFLD and control participants. For all subjects, there were no significant differences in age and sex between NAFLD and control subjects, indicating the study was an age- and sex-matched case-control study. Several risk factors for NAFLD were significantly different between the 2 groups: BMI, TG, TC, HDL, LDL, SUA, FPG, AST, ALT, BUN, ADP, RBP4 and CK18-M30 (*P* < 0.05).

**Table 1:** Clinical characteristics of the patients and control subjects Characteristic

characteristics	control	case	*p*
Number	303	317	
Male (%)	137 (45.21%)	149 (47.00%)	0.687
Age(years)	42.44 ± 10.05	42.92 ± 9.44	0.326
BMI(kg/m^2^)	24.54 ± 3.84	28.76 ± 3.86	< 0.001
FPG(mmol/L)	4.81 ± 0.82	5.48 ± 1.87	< 0.001
TG(mmol/L)	1.19 ± 0.69	2.12 ± 2.05	< 0.001
TC(mmol/L)	4.26 ± 1.38	4.60 ± 1.69	0.002
HDL(mmol/L)	1.38 ± 0.35	1.34 ± 1.41	< 0.001
LDL(mmol/L)	2.86 ± 0.78	3.08 ± 0.84	< 0.001
BUN(mmol/L)	4.82 ± 1.27	4.86 ± 1.22	0.408
SCr(μmol/L)	67.22 ± 18.02	68.18 ± 18.26	0.510
UA(μmol/L)	260.31 ± 302.96	86.40 ± 87.66	< 0.001
AST(U/L)	19.99 ± 7.39	23.67 ± 14.35	< 0.001
ALT(U/L)	23.33 ± 15.68	35.03 ± 27.81	< 0.001
ADP(ug/L)	813.71 ± 339.70	451.49 ± 134.18	< 0.001
RBP4(pg/ml)	126.00 ± 25.15	218.35 ± 136.57	< 0.001
CK18-M30(ng/L)	143.57 ± 70.85	246.25 ± 108.80	< 0.001
CK18-M65(ng/L)	199.72 ± 171.48	182.61 ± 153.05	0.196

*BMI* Body mass index, *TG* serum triglyceride, *TC* total cholesterol, *HDL* high-density lipoprotein, *LDL* low-density lipoprotein, *SUA* serum uric acid, *FPG* fasting plasma glucose, *AST* aspartate aminotransferase, *ALT* alanine aminotransferase, *BUN* blood urea nitrogen, *SCr* serum creatinine

### H-W equilibrium test

The results for the observed and expected values of the genotypes at the polymorphisms locus rs1260326, rs780094, rs2954021, rs2228603, rs58542926, rs12137855, and rs10883437 in the control group were in H-W equilibrium, indicating that the samples in these groups were representative of the population, as shown in Table 2.

**Table 2:** The association between the risk of NAFLD and the genetic polymorphisms

SNP	WT Ho/ Ht/ VR Ho		VR Ho vs WT Ho		Ht vs WT Ho		Dominant model		Recessive model		VR allele vs WT allele	
	Control	NAFLD	*P*	OR (95%CI)	*P*	OR (95% CI)	*P*	OR (95% CI)	*P*	OR (95% CI)	*P*	OR (95% CI)
rs1260326	115/145/43	103/149/65	0.03	1.69 (1.06–2.70)	0.09	1.15 (0.81–1.63)	0.15	1.27 (0.91–1.77)	0.04	1.56 (1.02–2.38)	0.04	1.27 (1.01–1.59)
rs780094	120/137/46	101/150/66	0.02	1.70 (1.08–2.70)	0.06	1.30 (0.92–1.85)	0.04	1.40 (1.01–1.95)	0.07	1.47 (0.97–2.22)	0.02	1.30 (1.04–1.63)
rs2954021	94/144/65	82/173/62	0.20	1.09 (0.69–1.73)	0.09	1.38 (0.95–1.99)	0.15	1.29 (0.91–1.83)	0.56	0.89 (0.60–1.32)	0.56	1.07 (0.85–1.34)
rs2228603	242/57/4	253/58/6	0.58	1.43 (0.40–5.15)	0.84	0.97 (0.65–1.46)	0.99	1.00 (0.68–1.49)	0.57	1.44 (0.40–5.16)	0.86	1.03 (0.73–1.46)
rs58542926	268/34/1	270/44/3	0.35	2.98 (0.31–28.81)	0.36	1.28 (0.80–2.07)	0.23	1.33 (0.83–2.13)	0.33	2.89 (0.30–27.89)	0.18	1.35 (0.87–2.09)
rs12137855	200/96/7	223/83/11	0.49	1.41 (0.54–3.71)	0.25	0.78 (0.55–1.10)	0.25	0.82 (0.58–1.15)	0.39	1.52 (0.58–3.97)	0.46	0.89 (0.67–1.20)
rs10883437	175/115/13	168/134/15	0.64	1.20 (0.56–2.60)	0.49	1.21 (0.88–1.68)	0.23	1.21 (0.88–1.67)	0.79	1.11 (0.52–2.37)	0.27	1.17 (0.89–1.53)

*OR* odds ratio; *VR* variant; *WT* wild-type; *Ht* heterozygote; *VR Ho*, variant homozygote; *WT Ho*, wide-type homozygote

### Association analysis

For rs1260326, the variant homozygote vs. wide-type homozygote (TT vs. CC: OR = 1.69, 95% CI = 1.06–2.70, *p* = 0.03), the recessive model (CC + CT vs. TT: OR = 1.56, 95% CI =1.02–2.38, *p* = 0.04), and the variant allele vs. wide-type allele (T vs. C: OR = 1.27, 95% CI = 1.01–1.59, p = 0.04), showed a significant difference between NAFLD and control participants. In addition, for rs780094, the variant homozygote vs. wide-type homozygote (TT vs. CC: OR = 1.70, 95% CI = 1.08–2.70, *p* = 0.02), the dominant model (CC vs. CT + TT: OR = 1.40, 95% CI =1.01–1.95, *p* = 0.04), and the variant allele vs. wide-type allele (T vs. C: OR = 1.30, 95% CI = 1.04–1.63, p = 0.02), showed a significant difference between NAFLD and control participants. However, logistic regression analyses revealed that the five polymorphisms (rs2954021, rs2228603, rs58542926, rs12137855 and rs10883437) were not associated with the risk of NAFLD (Table 2).

Table 3 also showed risk of NAFLD based on rs1260326 and rs780094 taking into consideration red meat, soda, egg, vegetables, fruits and soybean consumption. In the consumption group with once or less in a week, no significant associations were found between rs1260326 and rs780094 genotypes and risk of NAFLD. However, in the consumption group with twice or more in a week, taking C/C genotype group as reference, the rs1260326 T/T genotype group among the egg (OR = 1.73, 95% CI = 1.01–2.95, *p* = 0.046) and soybean (OR = 1.76, 95% CI = 1.07–2.90, *p* = 0.03) intakes demonstrated increased risk of NAFLD; the rs780094 T/T genotype group among the soda (OR = 1.70, 95% CI = 1.01–2.83, *p* = 0.04), egg (OR = 1.81, 95% CI = 1.07–3.06, *p* = 0.03) and soybean (OR = 1.74, 95% CI = 1.07–2.84, *p* = 0.03) intakes also demonstrated increased risk of NAFLD.

**Table 3:** Association of rs1260326 and rs780094 with Food Habits and Risk of NAFLD

Category	rs1260326				rs780094			
	Case	Control	OR (95% CI)	*P*-value	Case	Control	OR (95% CI)	*P*-value
Red meat								
(C/C) (≤1)	49	14	1.00		46	15	1.00	
(C/T) (≤1)	63	17	1.06(0.48–2.36)	0.89	64	14	1.49(0.66–3.39)	0.34
(T/T) (≤1)	29	7	1.18(0.43–3.27)	0.75	31	9	1.12(0.43–2.89)	0.81
(C/C) (> 1)	55	93	1.00		56	97	1.00	
(C/T) (> 1)	76	123	1.04(0.67–1.62)	0.85	76	118	1.12(0.72–1.73)	0.62
(T/T) (> 1)	34	34	1.69(0.95–3.02)	0.08	33	35	1.63(0.92–2.91)	0.1
Soda								
(C/C) (≤1)	26	2	1.00		27	2	1.00	
(C/T) (≤1)	48	2	1.85(0.25–13.88)	0.84	46	3	1.14(0.18–7.23)	0.34
(T/T) (≤1)	17	1	1.31(0.11–15.57)	0.88	18	0	–	–
(C/C) (> 1)	76	103	1.00		73	108	1.00	
(C/T) (> 1)	88	136	0.88(0.59–1.31)	0.52	91	128	1.05(0.70–1.57)	0.81
(T/T) (> 1)	47	38	1.68(1.00–2.82)	0.05	47	41	*1.70(1.01–2.83)*	*0.04*
Egg								
(C/C) (≤1)	27	13	1.00		28	14	1.00	
(C/T) (≤1)	51	14	1.75(0.72–4.26)	0.22	50	12	2.08(0.85–5.12)	0.11
(T/T) (≤1)	18	7	1.24(0.41–3.70)	0.7	18	8	1.12(0.39–3.22)	0.83
(C/C) (> 1)	76	97	1.00		73	101	1.00	
(C/T) (> 1)	88	127	0.88(0.59–1.33)	0.55	90	121	1.03(0.69–1.54)	0.89
(T/T) (> 1)	46	34	*1.73(1.01–2.95)*	*0.046*	47	36	*1.81(1.07–3.06)*	*0.03*
Vegetables								
(C/C) (≤1)	53	95	1.00		51	101	1.00	
(C/T) (≤1)	77	123	1.12(0.72–1.74)	0.61	79	115	1.36(0.87–2.12)	0.17
(T/T) (≤1)	28	40	1.24(0.41–3.70)	0.7	28	42	1.32(0.74–2.37)	0.35
(C/C) (> 1)	54	15	1.00		54	14	1.00	
(C/T) (> 1)	64	17	1.05(0.48–2.29)	0.91	63	18	0.91(0.41–1.99)	0.81
(T/T) (> 1)	36	3	3.33(0.90–12.35)	0.07	37	3	3.20(0.86–11.91)	0.08
Fruit								
(C/C) (≤1)	39	92	1.00		37	97	1.00	
(C/T) (≤1)	42	111	0.89(0.53–1.50)	0.67	43	105	1.07(0.64–1.80)	0.79
(T/T) (≤1)	14	35	0.94(0.46–1.95)	0.88	15	36	1.09(0.54–2.23)	0.81
(C/C) (> 1)	68	17	1.00		68	17	1.00	
(C/T) (> 1)	99	28	0.88(0.45–1.74)	0.72	99	26	0.95(0.48–1.89)	0.89
(T/T) (> 1)	50	8	1.56(0.63–3.91)	0.34	50	10	1.25(0.53–2.96)	0.61
Soybean								
(C/C) (≤1)	18	4	1.00		16	4	1.00	
(C/T) (≤1)	13	6	0.48(0.11–2.06)	0.32	15	6	0.62(0.15–2.66)	0.53
(T/T) (≤1)	7	3	0.52(0.09–2.93)	0.46	7	3	0.58(0.10–3.33)	0.54
(C/C) (> 1)	87	102	1.00		87	107	1.00	
(C/T) (> 1)	126	135	1.09(0.75–1.59)	0.64	125	127	1.21(0.83–1.76)	0.32
(T/T) (> 1)	57	38	*1.76(1.07–2.90)*	*0.03*	58	41	*1.74(1.07–2.84)*	*0.03*

OR (95% CI) and *P* values were obtained from logistic regression analysis. ≤1: Once or less in a week; > 1: Twice or more in a week; The numbers in italics indicated statistically significant values

### Haplotype analysis

To evaluate the correlations of the SNPs in GCKR, we performed haplotype analysis between NAFLD and healthy controls. There are total of four common haplotypes (> 1%) among controls. The haplotype CC, compared with the other three haplotypes, was demonstrated more frequently in both NAFLD and healthy controls. And using the most common haplotype as reference, a significant haplotype effect of rs1260326/T- rs780094/T was found (OR = 1.29, 95% CI: 1.03–1.62) (Table 4).

**Table 4:** Haplotypes of the GCKR gene with the risk of NAFLD

Haplotypes	Control frequency	Case frequency	OR (95% CI)	*P*-value
rs1260326/rs780094				
CC	0.607	0.549	1.00	–
TT	0.366	0.434	1.29(1.03–1.62)	0.027
CT	0.012	0.011	1.02(0.35–2.95)	0.970
TC	0.015	0.006	0.48(0.15–1.60)	0.230

OR (95% CI) and *P* values were obtained from logistic regression analysis

### Genotype of the two polymorphisms and the clinical characteristics of the patients

**Fig. 2:**
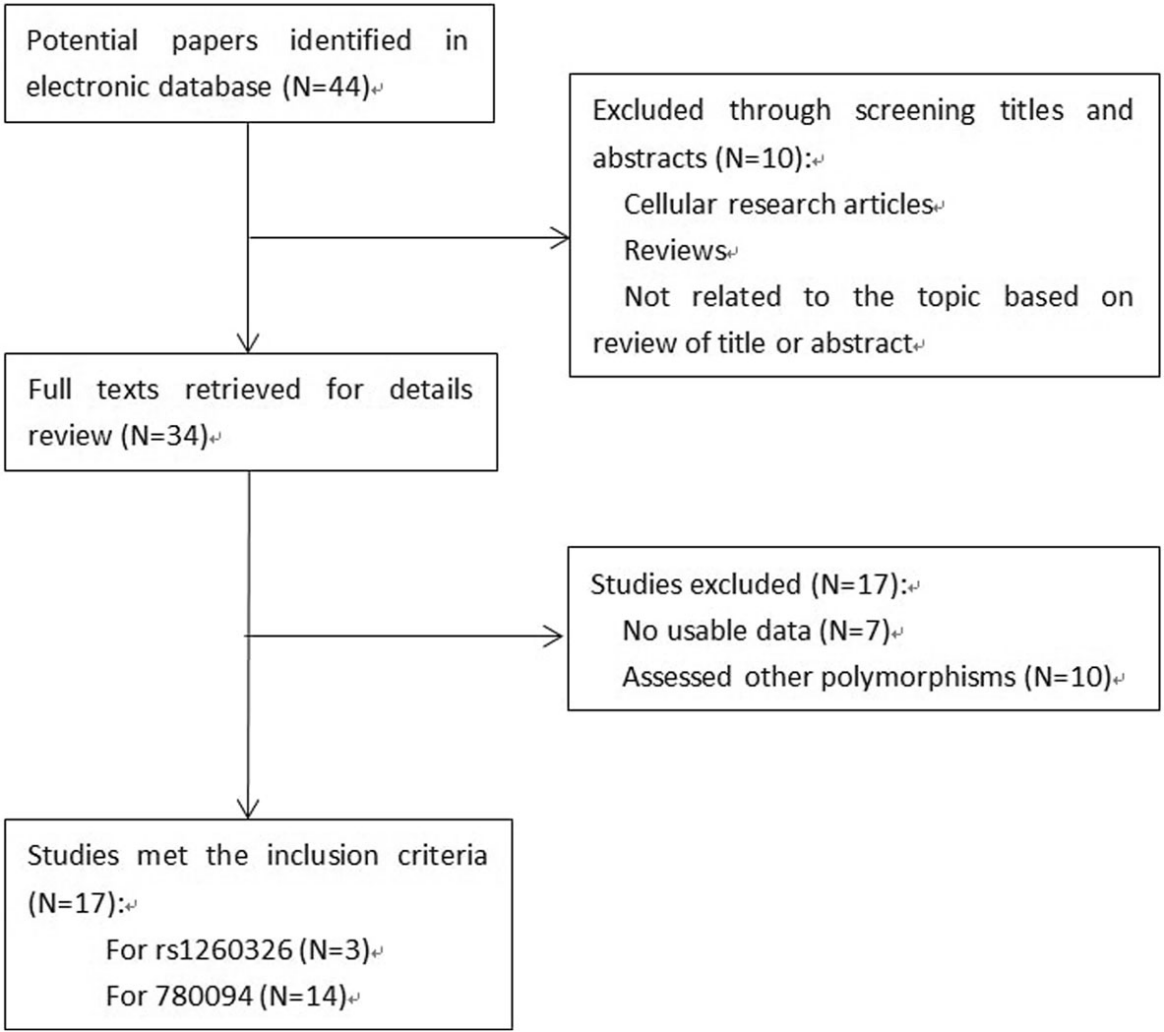
Flowchart of the study selection

To investigate whether there are clinical characteristics differences between the two significant polymorphisms (rs1260326 and rs780094) and each genotype, we calculated the clinical characteristics for each genotype. The genotypes of polymorphism rs1260326 include three genotypes of CC/CT/TT, so is rs780094. However, the differences of various clinical indicators among different groups were not statistically significant (*P* > 0.05) (Fig. 2).

### Meta-analysis

**Fig. 3:**
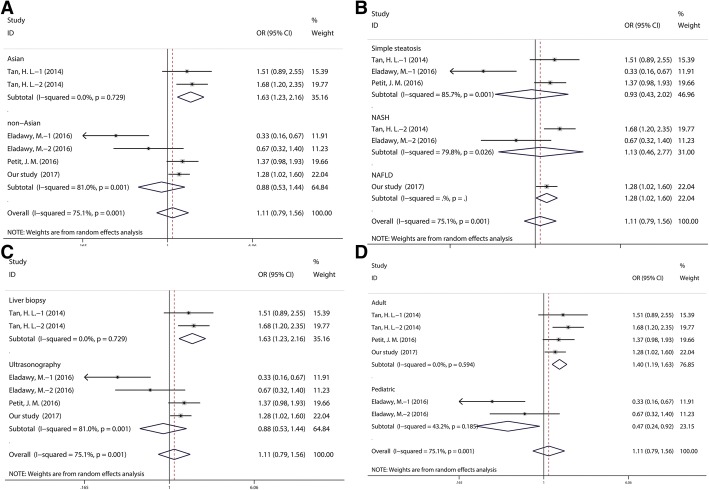
Forest plot of NAFLD susceptibility associated with rs1260326 (**a**-**d**) polymorphism at allele model (T vs. C)

Information from the eligible studies and our case-control study are shown in Table 5. Among the 4 case-control studies on rs1260326, including 712 NAFLD and 658 controls, 1 study only had allelic information. Therefore, allele-based model was applied in the meta-analysis and no significant association between the rs1260326 polymorphism and the risk of NAFLD was found for the overall analysis (OR = 1.11, 95%CI: 0.79–1.56). However, the heterogeneity is significant (I^2^ = 75.1%, *P* = 0.001). And subgroup meta-analysis by age has lowered the heterogeneity (for adult: I^2^ = 0, P = 0.001; for pediatric: I^2^ = 43.2%, *P* = 0.185). Furthermore, the adult subgroup and pediatric group showed significant association (OR = 1.40, 95%CI: 1.19–1.63 for the adult group, OR = 0.47, 95%CI: 0.24–0.92 for the pediatric group). Similarly, stratified analysis by ethnicity, liver disease, and NAFLD assessment showed significant associations existed among Asian group (OR = 1.40, 95%CI: 1.18–1.68), NAFLD group (OR = 1.28, 95%CI: 1.02–1.60) and liver biopsy group (OR = 1.63, 95%CI: 1.23–2.16) (Fig. 3) Additional file 1.

**Table 5:** Characteristics of Studies Included in this meta-analysis

Author	Year	Ethnicity	Liver disease	NAFLD assessment	Source of control	Age	Obesity	Sample size
rs1260326								
Tan, H. L.	2014	Asian	Simple steatosis/NASH	Liver biopsy	Hospital based	Adult	NA	342
Eladawy, M.	2016	non-Asian	Simple steatosis/NASH	Ultrasonography	Hospital based	Pediatric	NA	100
Petit, J. M.	2016	non-Asian	Simple steatosis	Ultrasonography	Hospital based	Adult	NA	308
Our study	2017	Asian	NAFLD	Ultrasonography	Hospital based	Adult	NA	620
rs780094								
Tan, H. L.	2014	Asian	Simple steatosis/NASH	Liver biopsy	Hospital based	Adult	Non-obese	342
Lin, Y. C.	2014	Asian	NAFLD	Ultrasonography	Population based	Pediatric	Obese	796
Dold, L.	2017	Caucasian	NAFLD	Ultrasonography	Hospital based	Adult	Non-obese	227
Lin, Y. C.	2016	Asian	NAFLD	Ultrasonography	Population based	Pediatric	Obese	827
Tai, C. M.	2016	Asian	Simple steatosis/NASH	Liver biopsy	Hospital based	Adult	Obese	177
Wang, X.	2016	Asian	NAFLD	Ultrasonography	Population based	Adult	Non-obese	763
Shang, X. R.	2015	Asian	Simple steatosis	Ultrasonography	Population based	Pediatric	Non-obese	1026
Kanth, V. V.	2014	Asian	NAFLD	Ultrasonography	Population based	Adult	Non-obese	306
Kitamoto, A.	2014	Asian	NAFLD	Liver biopsy	Population based	Adult	Non-obese	1552
Gorden, A.	2013	Caucasian	NAFLD	Liver biopsy	Hospital based	Adult	Obese	1055
Yang, Z.	2011	Asian	Simple steatosis	Ultrasonography	Population based	Adult	Non-obese	903
Speliotes,E.K	2011	Caucasian	NASH	Liver biopsy	Hospital based	Adult	Non-obese	5209
Dong, Q. Y.	2015	Asian	NAFLD	Ultrasonography	Hospital based	Adult	Non-obese	332
Song, X. C.	2016	Asian	NAFLD	Ultrasonography	Population based	Adult	Non-obese	384
Our study	2017	Asian	NAFLD	Ultrasonography	Hospital based	Adult	Non-obese	342

We found 14 case-control studies on rs780094, 9 more cases than were used in the most recently published meta-analysis in 2014 [34]. Therefore, we performed an updated meta-analysis to investigate the link between rs780094 and NAFLD. Among the 15 eligible studies including 4352 NAFLD and 10,168 controls in the current meta-analysis, 5 studies only had allelic information. Therefore, allele-based model was applied in the meta-analysis and a significant association between the rs780094 polymorphism and the risk of NAFLD was found for the overall analysis (OR = 1.21, 95%CI: 1.14–1.28). In a stratified analysis by ethnicity, liver disease, source of control, NAFLD assessment, age, and obese status, we further detected that all subgroups showed significant associations (OR = 1.21, 95%CI: 1.13–1.29 for the Asians group, OR = 1.20, 95%CI: 1.09–1.32 for the Caucasians group, OR = 1.18, 95%CI: 1.03–1.35 for the simple steatosis group, OR = 1.25, 95%CI: 1.12–1.40 for the NASH group, OR = 1.20, 95%CI: 1.11–1.29 for the NAFLD group, OR = 1.23, 95%CI: 1.13–1.33 for the hospital based group, OR = 1.19, 95%CI: 1.10–1.28 for the population based group, OR = 1.26, 95%CI: 1.16–1.36 for the liver biopsy group, OR = 1.16, 95%CI: 1.07–1.25 for the ultrasonography group, OR = 1.19, 95%CI: 1.12–1.27 for the adult group, OR = 1.27, 95%CI: 1.11–1.46 for the pediatric group, OR = 1.25, 95%CI: 1.11–1.41 for the obese group, and OR = 1.19, 95% CI: 1.12–1.27 for the Non-obese group, respectively) (Fig. 3).

**Fig. 4:**
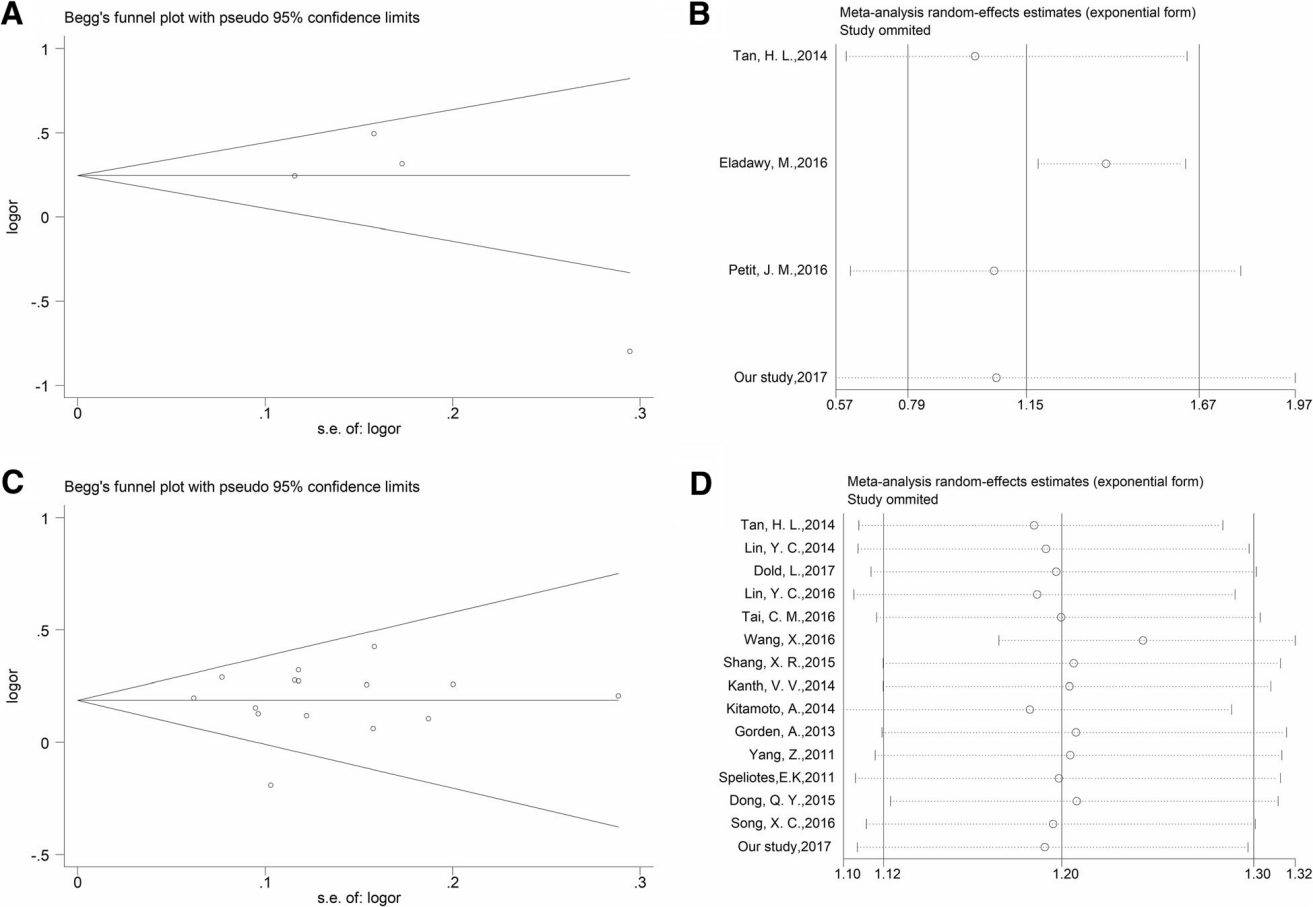
Funnel plot for the assessment of publication bias (**a**: rs1260326 and **c**: rs780094) and sensitivity analysis (**b**: rs1260326 and **d**: rs780094) in this meta-analysis

The meta-analyses on the two polymorphisms showed no publication bias by Begg’s funnel plot analysis (for rs1260326: *P* = 0.734; for rs780094: *P* = 0.921). Furthermore, sensitivity analysis also revealed that the conclusion was not biased by any individual study (Fig. 4).

## Discussion

Our results showed that the rs1260326 and rs780094 polymorphisms in the GCKR gene were significantly associated with NAFLD in Uygur population. The minor T allele of GCKR rs1260326 and rs780094 may increase the risk of NAFLD.

GCKR, a negative regulator of glucokinase, regulates phosphorylation of glucose, glycolysis, and fatty acid synthesis in the liver [35, 36]. A non-synonymous GCKR variant (rs1260326) encoding for the proline-to-leucine substitution at amino acid position 446 (P446L), seems to affects GCKR’s ability to negatively regulate glucokinase in response to fructose-6-phosphate, thereby constitutively activating hepatic glucose uptake [37], which would leads to decreased circulating fasting glucose and insulin levels. However, the corresponding increasing production of malonyl-CoA may accumulate hepatic fat which serves as a substrate for lipogenesis and blocks fatty acid oxidation [38]. Numerous studies have found the non-functional GCKR rs780094 variant is in strong linkage disequilibrium with rs1260326 (HapMap CEU r^2^ = 0.93, CHB r^2^ = 0.82) [21, 39, 40]. And our haplotype analysis has found the haplotype rs1260326/T−/rs780094/T, compared with rs1260326/C−/rs780094/C haplotype, showed significant effect with NAFLD.

However, inconsistent with the previous findings that GCKR rs1260326 and rs780094 were significantly associated with insulin, triglyceride and fasting plasma glucose levels [8, 21], we did not observe significant clinical characteristics differences between the two significant polymorphisms and each genotype, suggesting their potential interaction with environment, such as BMI, obesity and age [25]. However, we did not find significant associations between either rs2954021 near TRIB1, rs2228603 in NCAN, rs58542926 in TM6SF2, rs12137855 near LYPLAL1, or rs10883437 near CPN1 and NAFLD in Uygur population, indicating these variants may not the causal variants associated with NAFLD in Uygur.

Additionally, our study revealed that increased risk estimates were observed for interaction of rs1260326 T/T genotype with egg and soybean and rs780094 T/T genotype with soda, egg and soybean in the consumption group with twice or more in a week. Thus, understanding the mechanism of rs1260326 T/T and rs780094 T/T genotypes with soda, egg and soybean interaction will require further studies. Moreover, the correlation among ADP, RBP4, CK18, rs1260326, rs780094 and NAFLD was investigated in this study. The results showed a negative correlation between ADP and NAFLD; a positive correlation between RBP4 and NAFLD; a positive correlation between CK18-M30 and NAFLD. However, the differences of ADP, RBP4, CK18-M30 and CK18-M65 among different genotypes of rs1260326 and rs780094 in NAFLD patients were not statistically significant, which suggested the ADP, RBP4 and CK18-M30 might not be functioned by rs1260326 and rs780094 in the formation of NAFLD, and further fine-mapping studies in the susceptible region of the variants and more NAFLD-related serum cytokines are needed.

In the present study, we further do the meta-analysis for the two significant polymorphisms (rs1260326 and rs780094). The current meta-analysis includes 17 studies comprised of 4603 cases and 10,325 controls. The pooled results revealed that the GCKR rs780094 polymorphism was associated with increased risk of NAFLD. Moreover, stratified analysis by ethnicity, liver disease, source of control, NAFLD assessment, age, and obesity has demonstrated all subgroups were significantly correlated with increased risk of NAFLD, suggesting GCKR rs780094 may have a high effect on NAFLD incidence. Unexpectedly, we found no significant associations between GCKR rs1260326 and risk of NAFLD for the overall results with significant heterogeneity (I^2^ = 75.1%, *P* = 0.001). Of note, when stratified by age, the heterogeneity was lower among the two subgroups with adverse significant associations, suggesting age difference may exist on GCKR rs1260326. Additionally, significantly increased risk of NAFLD among Asian population and liver biopsy groups were found in GCKR rs1260326. However, the limited sample size may have underestimated subtle effects of the genetic variant, and the above conclusions should be carefully considered.

Nevertheless, several limitations need to be addressed. First, the NAFLD patients and controls for rs1260326 were enrolled from hospitals which may not represent the general population. Second, only published studies in English or Chinese were enrolled and may lead to a selection bias in our meta-analysis. In addition, the polymorphisms investigated in our study may not be sufficiently comprehensive about genetic variability in these genes. And further fine-mapping studies in the susceptible region of the variants are needed. At last, further studies are warranted to confirm our findings, particularly the potential effects of gene-gene and gene-environment interactions should be considered.

In summary, our case-control and meta-analysis demonstrates that the frequency of the GCKR rs780094-T allele is significantly increased in NAFLD cases compared with controls, as well as stratified analysis by ethnicity, liver disease, source of control, NAFLD assessment, age, and obese status. Furthermore, the GCKR rs1260326-T allele is significantly associated with NAFLD among Asian, liver biopsy, adult and pediatric groups. Moreover, the rs1260326 and rs780094 T/T genotype are significantly associated with food habits, such as soda, egg, and soybean.

## Additional file

Additional file 1: Supplement figure 1. Forest plot of NAFLD susceptibility associated with rs1260326 (A-D) polymorphism at allele model (T vs. C). (JPEG 2020 kb)
